# The prevalence and root causes of delay in seeking healthcare among mothers of under five children with pneumonia in hospitals of Bahir Dar city, North West Ethiopia

**DOI:** 10.1186/s12887-019-1869-9

**Published:** 2019-12-09

**Authors:** Getasew Mulat Bantie, Zemene Meseret, Melkamu Bedimo, Abebayehu Bitew

**Affiliations:** 1 GAMBY Medical and Business College, department of Public Health, Bahir Dar, Ethiopia; 2 Felegehiwot comprehensive specialized hospital, Bahir Dar, Ethiopia; 3 Department of Biostatistics and Epidemiology, Bahir Dar university, Bahir Dar, Ethiopia

**Keywords:** Pneumonia, Delay in seeking healthcare, Associated factor, Children, Ethiopia

## Abstract

**Background:**

Globally pneumonia is the leading cause of under-five child mortality. Several risk factors for pneumonia mortality have been identified, including delay in seeking health care. For successful reduction of delay in seeking healthcare, further evidence is crucial on its magnitude and factors associated with it in the country particularly in the study area. Therefore, this study aimed to determine the prevalence and root causes of delay in seeking health care among mothers of under-five children with pneumonia in hospitals of the Bahir Dar city, 2019.

**Methods:**

A hospital-based cross-sectional study was conducted from March 15 to May 15, 2019 among 356 mothers of under-five children with pneumonia in hospitals of the Bahir Dar city. The study participants were selected by using a stratified sampling technique and data was collected through face to face interview. Binary logistic regression was used to identify the associated factors of delay in seeking healthcare. The P - value < 0.05 was considered statistically significant. Associations between outcome and exposure variables were expressed by the adjusted odds ratio with a 95% confidence interval (CI).

**Results:**

A total of 356 mothers participated in the study yielded a response of 89.4%. The proportion of delay in seeking health care was 48.6%. Rural residence (AOR = 2. 3, 95% CI: 1.1, 4.9, seek healthcare in a governmental hospital (AOR = 3. 3, 95% CI: 1.8, 6.1), health care decision by mothers (AOR = 2. 9, 95% CI: 1.6, 5.4), poorest household (AOR = 2. 8, 95% CI: 1.1, 7.2), using self-medication (AOR = 7. 5, 95% CI: 3.8, 14.7), using traditional medicine before healthcare-seeking (AOR = 2. 7, 95% CI: 1.4, 5.1), and no information about early healthcare-seeking for childhood pneumonia treatment (AOR = 5. 1, 95% CI: 2.8, 9.1) were the identified determinants significantly associated with delay in seeking healthcare among mothers of under-five children with pneumonia.

**Conclusion:**

This study showed that nearly half of the mothers delayed in seeking healthcare. Rural residence, healthcare seeking at government hospitals, healthcare decision by mothers, poorest household, using self-medication, using traditional medicine before health care seeking, and lack of information about early healthcare-seeking were factors associated with a delay in seeking healthcare for under-five children with pneumonia. Hence, the government and other concerned stakeholders should give due emphasis to tackle on the identified causes of delay in seeking health care for the under five children with pneumonia.

## Background

Pneumonia is an infection of the lungs that is most commonly caused by bacteria or viruses [1]. Worldwide, pneumonia is the primary cause of morbidity and mortality among children less than five years of age [2]. It is accounted for nearly 16% of the 5.6 million under-five deaths in 2016 [3].

In Ethiopia, pneumonia is one of the leading causes of death in under-five children [4, 5]. About 13% of pneumonia cases are severe enough to require hospitalization, and 8.7% of these are severe enough to be life-threatening [4, 5]. The World Health Organization (WHO) and UNICEF reported that promptly seeking healthcare with an appropriate healthcare provider is one of the most important steps to saving the life of a child from pneumonia. Thus, prompt and appropriate care-seeking for childhood pneumonia has implications both in terms of child survival as well as the expenditures at the household and the health system levels [6]. However, worldwide, only 60% of children receive the necessary help and care. In sub-Saharan Africa, where most pneumonia deaths occur, only 40% of children seek care [7].

The fight against pneumonia-related deaths in children relies on the activity of pneumonia prevention, including prompt health care seeking [7]. Delay in healthcare-seeking contributes to a large number of pneumonia deaths in developing countries [8]. According to a WHO report, 70% of child mortality is related to inadequate or delay in seeking health care and can be prevented by seeking health care earlier [9].

In Ethiopia, health care seeking behavior is poor and only a small proportion of children receive appropriate treatment [10]. Factors such as residence, educational status of caregivers, marital status of caregivers knowledgeability on danger signs, number of symptoms experienced by the child, and perceived severity of the illness have been associated with delay in health care seeking [11, 12]. Despite some studies in some parts of the country about health care seeking behavior for pneumonia treatment, nothing has been done and still there is an information gap in the Bahir Dar City hospitals. Therefore, this study aimed to determine the prevalence and root causes of delay in seeking health care among mothers of under-five children with pneumonia in hospitals of the Bahir Dar city to design appropriate interventions for the improvement of child health in the study area.

## Methods

### Study area

This hospital-based cross-sectional study was conducted in Bahir Dar city hospitals from March 15up to May 15, 2019. Bahir Dar city is the capital city of the Amhara Region, which is 565Km from Addis Ababa, the capital city of Ethiopia. In the city, there are two governmental (Felegehiwot referral and Addis Alem primary hospital) and three private hospitals (GAMBY Teaching Hospital, Adinas and Dream Care general hospital). These five hospitals provide services to around 7 million people from the surrounding area. These people are visiting the hospitals from different geographical areas (Dega, woynadega and kola), of the region for better health services. The Felegehiwot referral hospital is one of the comprehensive, specialized governmental hospitals providing trainings in internal medicine, surgery, gynecology and obstetrics and pediatrics specialties for advanced education. This hospital has the pediatrics outpatient and inpatient departments, including newborn intensive care unit. This hospital provides services for about 4 million communities coming from more than 500 km distance. The other four hospitals are primary, general hospitals having pediatrics inpatient and outpatient departments. All these five hospitals are holding ‘the lion part’ for the health care delivery of the region [13].

### Population

Mothers of under-five children with pneumonia who seek health care in hospitals of the Bahir Dar city were the source population, while mothers of under-five children with pneumonia who seek health care in the study period were the study population.

### Eligibility criteria

Mothers of under-five children with pneumonia who seek health care in hospitals of the Bahir Dar city were included, while mothers of under-five children who visited the hospitals for appointment/follow up of pneumonia treatment were excluded from the study.

### Sample size determination and sampling procedure

**Fig. 1:**
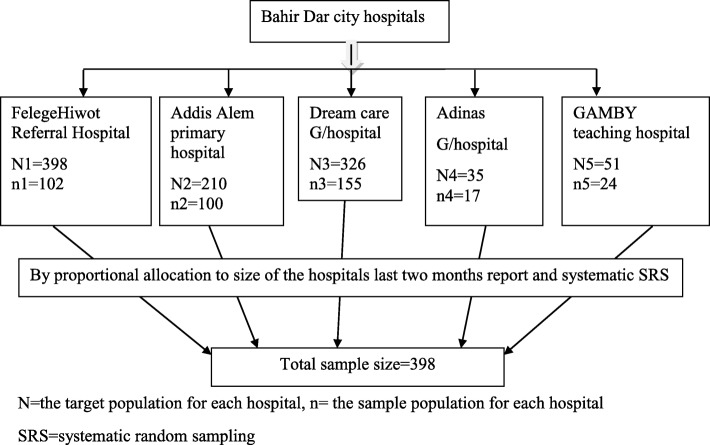
Schematic presentation of sampling procedures at Bahir Dar city hospitals, 2019

The sample size was determined by a single population proportion formula by the assumption that a 95% confidence interval, 5% margin of error and 52.1% of delay in seeking the healthcare [14]. Then, by adding a 10% non-response rate, the total sample size was 398. Mothers of under-five children with pneumonia visiting the Bahir Dar city hospitals were selected. Proportion to size allocation was made to determine the required sample size from each hospital based on the previous two months under-five pneumonia case report, which were 836. Systematic random sampling technique was used to select 398 mothers of under-five children with pneumonia. (Fig. 1)

### Study variables

#### Dependent variable

Delay in seeking health care for pneumonia.

#### Independent variables

Socio-demographic variables: Sex and age of children/mother, residence, marital status, religion, educational status, number of family members, the number of under-five children in the household.

Socioeconomic variables: Wealth, occupation, perception of medical cost, community health insurance.

Health facility related variables: Type of nearest health facility, mother preference on the type of health facility, distance to the nearest health facility.

Behavioral and clinical related variables: Knowledge, decision-maker to seek health care, use of traditional medicine, self-medication, use of holy water, signs/symptoms which leads to health care seeking, information on early health care seeking for childhood pneumonia treatment.

### Operational definitions and measurements

#### Delay in seeking health care

Seeking healthcare from health facilities after 24 h from the recognition of cough/fast breathing [14].

#### Promptness in seeking health care

Healthcare is seeking from health facilities within 24 h from the recognition of sign and symptom of pneumonia.

#### Self-medication

Purchasing and utilizing medicine from a pharmacy or shops without a prescription.

#### Traditional medicine

Experience-based knowledge and practice applied to treat patients with apparent illness and sickness by traditional healers, wogesha, herbalists, and magicians.

#### A scoring system for knowledge assessment

A total of 14 questions that measure mother’s knowledge, the score for each is given as 1 for the correct answer, and 0 for a not sure or wrong answer. The total was 14 and mothers’ knowledge is categorized as good knowledge (> 11) and poor knowledge (< 11) [15].

#### Wealth index

Wealth was determined based on consumer goods and household assets. Households are then ranked, from lowest to highest score. Then those scores were separated into Quintiles; each representing 20% of the population. Therefore, those in the highest quintile may not be “rich” but they are of higher socioeconomic status than 80% of the participants in this study [16].

### Data collection process and quality control

The pre-tested semi - structured questionnaire was prepared in English and translated to Amharic (local language), and the Amharic version was used for the interview. The data collection tool was comprised of demographic, socioeconomic, health facility, behavioral and clinical related factors. The pre-test was done for 5% of the sample size at Merawi primary hospital. The one-day training was given for six pediatric OPD working nurses, one from each hospital. The data were collected at pediatrics OPD after the child was diagnosed with pneumonia by physicians. The assigned nurses collected the data from the mothers by face to face interview. Continuous supervision of the data collection process was carried out to assure the quality of the data. Finally, the collected data were carefully checked daily for completeness.

### Data processing and analysis

Data were coded and entered into the Epi-data version 3.1 and then exported to SPSS version 23 for analysis. Both descriptive and inferential statistical analysis was employed. Summary statistics such as percentages were computed and odds ratios were calculated with 95% confidence intervals. A binary logistic regression model was used to test associations between each independent variable with the outcome variable. The principal component analysis was used to manage the wealth index. Variables significantly associated during simple binary logistic regression at *p*-value < 0.2 were considered as candidate variables for multiple binary logistic regression. Those variables which were significant in multiple binary logistic regression analysis of p-value < 0.05 were identified as an associated factor of delay in seeking healthcare.

### Ethical consideration

Ethical clearance obtained from the Bahir Dar University College of Medicine and Health Science Institutional Review Board and Permission letter was received from the study hospitals before preceding data collection. The study participants were informed about the purpose of the study and their right to participate or to withdraw the study at any time they want. A signed written consent was obtained from each study participant. The confidentiality of the information obtained was assured by coding and locking the data in a secure place.

## Results

### Socio-demographic characteristics of the respondents

In this study, a total of 356 study participants completed the interview yielded a response of 89.4%. Among these 284(79.8%) were from urban residences. One hundred forty (36.9%) had their children aged between 1 to 12 months with, mean (+SD) age of 22.3(+ 15) months and more than half 192(53.9%) of them were male children.

Most of the study participants 231 (64.9%) were aged between 25 to 34 years, with a mean (+SD) age of 30 (+ 5.7) years. A majority, 350 (98.4%) were married. 135 (37.9%) held tertiary levels of education and 74 (20.8%) had no formal education. From the study participants, 300 (84.3%) were of an orthodox religion. Most of the under-five children (330, 92.7%) were residing within the household of both parents. A majority of the mothers (254, 71.3%) has family sizes of two to five. The most commonly reported occupations were housewife (143, 40.2%). Sixty-eight mothers were the poorest. **(**Table 1**).**

**Table 1:** Socio-demographic characteristics of mothers and child related to delay in seeking healthcare in Bahir Dar city, North West Ethiopia, 2019

Variable	Category	Frequency	Percent
Sex of the child	Male	192	53.9
	Females	164	46.1
Age of the child in months	0–12	133	37.4
	13–24	93	26.1
	25–36	58	16.3
	37–48	55	15.4
	49–59	17	4.8
Age of caregiver	16–24	47	13.2
	25–34	231	64.9
	35–44	74	20.8
	> 45	4	1.1
Residence	Urban	284	79.8
	Rural	72	20.2
Marital status of caregivers	Married	350	98.4
	Divorced	3	0.8
	Single	3	0.8
Religion of caregiver	Orthodox	300	84.3
	Muslim	39	11.0
	Protestant	16	4.5
Educational status	Unable to read and write	72	20.8
	Primary education	62	17.4
	Secondary education	85	23.9
	Diploma and above	135	37.9
The child lives with whom	With a single parent	26	7.3
	With both parents	330	92.7
Number of under-five children in the Household	1	131	36.8
	> 1	243	63.2
Family size	2–5	254	71.3
	> 5	102	28.7
Occupation	Housewives	143	40.2
	Merchants	49	13.8
	Students	9	2.5
	Government employees	71	19.9
	NGO employees	17	4.8
	Farmers	59	16.6
	Unemployed	4	1.1
	Others	4	1.1
Wealth index	Poorest	68	19.1
	Poor	71	19.9
	Medium	74	20.8
	Rich	63	17.7
	Richest	80	22.5

### Health facility, behavioral and clinical related factors

Even though there were nearby hospitals, 59 % of mothers from the government and 26 % mothers from the private nearby hospitals were delayed in seeking health care for their child. Sixty four percent of mothers preferring to seek health care from the government hospitals were delayed while, 34 % of the private hospitals. Forty nine percent of mothers who spent less than an hour to arrive to the nearby health facility were delayed for the service. More than two-third of the mothers who did not get information on health care for pneumonia were delayed for the service. Fifty-five percent of mothers who made the decision for the healthcare by themselves were delayed for the health care service.**(**Table 2**).**

**Table 2:** Health facility, behavioral and clinical-related characteristics of respondents in Bahir Dar city, North West Ethiopia, 2019

Variable	Category	Health seeking		X^2^ at 95% CI
		Prompt	Delay	
Type of hospital closer to you	Government	91	130	X^2^ = 24.40
	Private	121	43	
Type of hospital, mothers prefer to seek healthcare	Government	62	110	X^2^ = 31.42
	Private	121	63	
Reasons to prefer private hospitals	Comprehensive examination	57	21	X^2^ = 7.10
	Low waiting time	37	12	
	Treatment is effective	19	9	
	Because they are near	4	6	
	Because they are respectful	7	3	
	Necessary medications are available	3	3	
	Always open/early open			
Reasons to prefer government hospitals	Because they are near	23	48	
	They don’t charge too much	10	37	
	Because they are respectful	2	14	
	Comprehensive examination	7	8	
	Always open/early open	8	7	
	Treatment is effective	0	3	
	Necessary medication availability	1	1	
Time taken to arrive to the nearest health facility on foot	< 60 min	171	167	X^2^ = 1.76
	> 60 min	12	6	
The severity of pneumonia (diagnosed at Pediatric OPD)	Severe pneumonia	21	51	X^2^ = 17.86
	Non-severe pneumonia	162	122	
Mothers reasons for a delay in seeking healthcare	The disease is resolved by itself	0	53	
	Self-medication	0	32	
	Transportation difficulties	0	13	
	Use of holy water at home	0	15	
	Use of traditional medicine	0	17	
	Lack of money	0	16	
	The illness was mild	0	28	
Information on healthcare seeking for pneumonia	No	37	105	X^2^ = 60.76
	Yes	146	68	
Mothers reason to seek healthcare promptly	Previous information on healthcare seeking on childhood pneumonia	101	0	
	Symptom worsen	63	0	
	Previous complication experience of delay	19	0	
The main source of information	At health center/hospital	83	35	
	Health extension workers	12	10	
	During community conversation	24	7	
	Neighbor	0	2	
	Media TV/Radio	25	11	
	Other^#^	2	3	
Symptoms lead the mother to seek healthcare	Cough/fast breathing	135	121	X^2^ = 5.97
	Fever	33	24	
	Chest-in drawing	2	5	
	Has a danger sign*	13	23	
Decision-maker to seek healthcare	Mother	89	107	X^2^ = 7.37
	Father	11	11	
	Both parents	72	47	
	Grandparents	7	6	
	Other^#^	4	2	
Perception of mothers on the cost of childhood pneumonia treatment	Easy to pay	56	91	X^2^ = 18.14
	Difficult to pay	123	78	
	Very difficult to pay	4	4	
Community health insurance	Yes	30	39	X^2^ = 2.15
	I don’t have	153	134	
knowledge on prompt health care seeking	Poor knowledge	131	154	X^2^ = 16.92
	Good knowledge	52	19	

Other^#^-relatives, peers; danger signs*-convulsion or lethargic/unconscious or vomiting everything.

### The proportion of delay in seeking healthcare

**Fig. 2:**
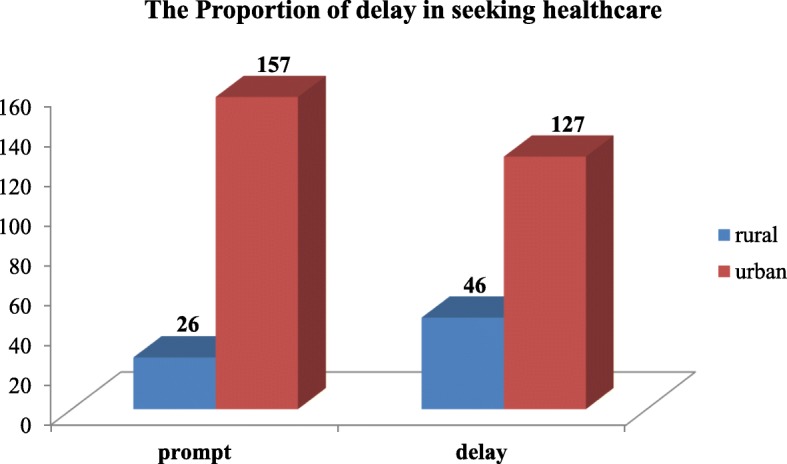
Proportion of delay in seeking health care among urban and rural mothers for under-five children with pneumonia, Bahir Dar, Ethiopia, 2019

From the total of 356 mothers seeking health care for a child with pneumonia, 173 (48.6, 95% C.I: 45.8–54.7) were delayed in seeking healthcare. Delay in seeking health care among mothers from rural residence was 46(63.9%), with 127 (44.7%) with delay for mothers of urban residence. (Fig. 2)

### Factors associated with delay in health care-seeking

Multivariable logistic regression analysis was conducted to identify the root causes of delay in seeking health care among mothers of under five children with pneumonia in hospitals of the Bahir Dar city, northwest Ethiopia. At multivariable logistic regression model residence of mothers, the decision-maker to seek health care, type of hospital mothers seek to the healthcare, wealth index, using traditional medicine, self-medication, and information on early healthcare-seeking associated with a delay in seeking health care at p-value of 0.05.

Residence of mothers was a significantly associated factor with delay in seeking healthcare. Accordingly, mothers from rural residence had about 2.3 times higher odds of delay in seeking health care (AOR = 2. 3, 95% CI: 1.1, 4.9) compared to urban. Similarly, mothers who prefer health care in a governmental hospital had about 3.3 times higher odds of delay in seeking health care for childhood pneumonia (AOR = 3. 3, 95% CI: 1.8, 6.1) compared to private hospitals.

A decision-maker at the household level has a significant role in the healthcare-seeking behavior. Healthcare is seeking decision for childhood pneumonia made by the mother had about 2.9 times higher odds of delay in seeking health care (AOR = 2. 9, 95% CI: 1.6, 5.4) compared to a decision made by both parents.

Mothers from the poorest households had about 2.8 times higher odds of delay in seeking health care (AOR = 2. 8, 95% CI: 1.1, 7.2) compared to mothers with the richest households.

Mothers using self-medication for an under-five child with pneumonia had about 7.5 times higher odds of delay in seeking health care (AOR = 7. 5, 95% CI: 3.8, 14.7) compared to those not using such medications. Mothers who used traditional medicine before healthcare-seeking at home for a child with pneumonia had about 2.7 times higher odds of delay in seeking health care (AOR = 2. 7, 95% CI: 1.4, 5.1) than those not using traditional medicine. Mothers who had no information about early healthcare-seeking for childhood pneumonia treatment had about 5 times higher odds of delay in seeking health care (AOR = 5. 1, 95% CI: 2.8, 9.1) than that of mothers who had information about early healthcare seeking. **(**Table 3**).**

**Table 3:** Factors associated with the delay in seeking health care among mothers of under-five children with pneumonia, Bahir Dar, North West Ethiopia, 2019

Variable	Category	Health seeking		COR (95%CI)	AOR (95% CI)
		Prompt	Delay		
Residence	Rural	26	46	2.2 (1.3, 3.7)	2.3 (1.1, 4.9)*
	Urban	157	127	1.00	1.00
Type of hospital mothers seek	Governmental	62	110	3.4 (2.2, 5.3)	3.3 (1.8, 6.1)***
	Private	121	63	1.00	1.00
Decision-maker to seek healthcare	Others	4	2	0.8 (0.1, 4.4)	0.8 (0.1, 6.8)
	Father	11	11	1.5 (0.6, 3.8)	0.9 (0.3, 2.9)
	Mother	89	107	1.8 (1.2, 2.9)	2.9 (1.6, 5.4)**
	Grandparents	7	6	1.3 (0.4, 4.2)	0.5 (0.1, 2.6)
	Both parents	72	47	1.00	1.00
Wealth index	Poorest	20	48	5.9 (2.9, 12.1)	2.8 (1.1,7.2)*
	Poor	33	38	2.8 (1.5, 5.6)	2.3 (0.9, 5.6)
	Medium	34	40	2.9 (1.5, 5.6)	1.8 (0.8, 4.2)
	Rich	39	24	1.5 (0.8, 3.1)	1.4 (0.6, 3.4)
	Richest	57	23	1.00	1.00
Using traditional medicine	Yes	33	64	2.7 (1.6, 4.3)	2.7 (1.4, 5.1)**
	No	150	109	1.00	1.00
Self-medication	Yes	19	70	5.9 (3.3, 10.3)	7.5 (3.8, 14.7)***
	No	150	109	1.00	1.00
Information on early healthcare-seeking	No	37	105	6.1 (3.8, 9.8)	5.1 (2.8, 9.1)***
	Yes	146	68	1.00	1.00

*statistically significant at *P* < 0. 05, **P < 0. 01, ***P < 0. 001

## Discussion

The present study revealed that the percentage of mothers delayed to seek health care for their pneumonia ill under-five children attending at Bahir Dar city hospitals was 48.6% (95%CI:45.8,54.7). This finding was lower than the studies conducted in Kenya (62.1%) and Tanzania (55.4%) [14, 17]. The possible explanation might be a difference in socio- demographic characteristics like study setting and lifestyle.

The study also identified the maternal reasons of delay for seeking health care for their pneumonia ill under-five children. These were initiating of self-medication, use of traditional medicine, lack of money, use of holy water and hoping that the disease could resolve by its own. This finding was supported by study findings in Uganda and Peru [18–20].

Seventy-eight percent of the mothers who initiated self-medication before visiting the physician at the hospital were delayed to seek healthcare. This finding was supported by other studies done in Rwanda and Uganda, where self-medication was shown to interfere with prompt health care seeking from hospitals [18, 21]. Similarly, 66 % of mothers who used traditional medicine were delayed to seek healthcare. This proportion was very high compared to the study conducted in Kenya, 2.3% [14]. This might be related to the cultural differences of the communities.

Seventy-one percent of mothers from the poorest household were delayed to seek healthcare. This result is supported by studies conducted in Nigeria and Guinea [22, 23].

Mothers from rural residence the odds of delay in seeking health care for pneumonia were 2.3 times higher compared to urban areas. This study is supported by other studies in Kenya and Ethiopia [24, 25]. The possible justification for this might be that urban residents could have better information access and awareness of pneumonia; as a result, this may encourage them to seek healthcare early for their under-five children. It is also the urban mothers might have money or cash to pay for health care services. However, rural households might not have money or cash for treatment; rather they get it by selling their crops and animals. This might take time and could be the possible cause of delay in seeking health care for rural mothers.

A mother who seeks health care from government hospitals the odds of delay in seeking health care for pneumonia for their under-five children were 3.3 times higher compared to private hospitals. This finding is supported by a study conducted in central Ethiopia [20, 26]. This could be mothers might not be satisfied with the services at government hospitals in the previous visit for their household healthcare.

Those mothers who used self-medication at home for childhood pneumonia treatment the odds of delay in seeking health care were about 7.5 times higher compared to mothers who did not use self-medication. This finding was supported by study findings in Pakistan and Rwanda [21, 26]. The possible justification for this might be the mothers may get the drugs from shops or collect from their neighbors’ which leads to wrong medication and the child may be exposed to overdose. As a result, this might create double jeopardy for the child.

Mothers who used traditional medicine for a child with pneumonia the odds of delay in seeking health care were 2.7 times higher compared to those who did not. This result is supported by a study conducted in Rwanda [21].

A decision made by a mother the odds of delay in seeking health care were 2.9 times higher compared to decision made by both parents. This study was in line with studies conducted in Kenya and Nigeria [23, 24]. This could be the mothers were depending on their husband for the cost of the healthcare services, which might enforce them to delay health care seeking for their child.

Mothers from the poorest households the odds of delay in seeking health care were 2.8 times higher compared to mothers from the richest households. This result is supported by studies conducted in Nigeria and Guinea [22, 23].

In this study, lack of information on early healthcare-seeking the odds of delay in seeking health care was about five times higher compared to those who had information. This result was supported by a study conducted in Ethiopia [27]. If mothers are aware of the advantage of early health care seeking, they are more likely to seek health care promptly for childhood pneumonia treatment.

## Conclusion

This study showed that nearly half of the mothers were delayed in seeking health care for childhood pneumonia treatment.

The finding of this study identified that rural residence, healthcare seeking at government hospitals, healthcare decision by mothers, poorest household, using self-medication, using traditional medicine before health care seeking, and lack of information about early healthcare-seeking were factors associated with a delay in seeking healthcare for under-five children with pneumonia. Hence, the government and other concerned stakeholders should give due emphasis to tackle on the identified causes of delay in seeking health care for the under five children with pneumonia.

### Recommendations

#### The regional health bureau

- Should better strengthen the regulations of un-prescribed medications and the use of traditional medicine in the region.
- Should provide exempted health service for under-five children to improve early health care seeking practice of mothers for childhood pneumonia
- Should better disseminate information via the mass media about the complications of delay in seeking health care.

#### Health facilities and partners

- Should better create awareness for mothers about the importance of prompt health care seeking.
- Should strengthen the health extension program via house to house visits and active surveillance to early detect the pneumonia cases from the community.
- Government health facilities should improve the quality of the healthcare services to the community for mothers to seek the service.
- Health facilities should improve healthcare provision approach of childhood pneumonia treatment, especially for governmental health facilities.

#### The researchers

- Further studies with strong evidence should be conducted.

## Data Availability

The data can be accessed from the corresponding author through the following address getasewmulat@gmail.com. The data will be accessed for research purpose and this is because, during the ethical clearance process, we agree with the Institutional review board of Bahir Dar University to keep the confidentiality of the data set.

## Abbreviations

ALRTI: Acute Lower Respiratory Tract Infection
AOR: Adjusted odds ratio
CI: Confidence Interval
DRC: Democratic Republic of Congo
OPD: Outpatient department
SPSS: Statistical Package for Social Scientist
SRS: Systematic random sampling
